# Antidepressant Effects of NSAIDs in Rodent Models of Depression—A Systematic Review

**DOI:** 10.3389/fphar.2022.909981

**Published:** 2022-06-08

**Authors:** Cecilie Bay-Richter, Gregers Wegener

**Affiliations:** Translational Neuropsychiatry Unit, Department of Clinical Medicine, Aarhus University, Aarhus, Denmark

**Keywords:** depression, animal model, behaviour, non-steroidal anti-inflammatory drug, neuroinflammation

## Abstract

In recent years much focus has been on neuroimmune mechanisms of depression. As a consequence, many preclinical and clinical trials have been performed examining potential antidepressant effects of several anti-inflammatory drugs. The results of such trials have been varied. With the current manuscript we wished to elucidate the effects of non-steroidal anti-inflammatory drugs (NSAIDs) on depressive-like behaviour in rodent models of depression by performing a systematic review of the available literature. We performed a systematic literature search in PubMed for rodent models of depression where NSAIDs were administered and a validated measure of depressive-like behaviour was applied. 858 studies were initially identified and screened using Covidence systematic review software. Of these 36 met the inclusion criteria and were included. The extracted articles contained data from both rat and mouse studies but primarily male animals were used. Several depression models were applied and 17 different NSAIDs were tested for antidepressant effects. Our results suggest that stress models are the best choice when examining antidepressant effects of NSAIDs. Furthermore, we found that rat models provide a more homogenous response than mouse models. Intriguingly, the use of female animals was only reported in three studies and these failed to find antidepressant effects of NSAIDs. This should be explored further. When comparing the different classes of NSAIDs, selective COX-2 inhibitors were shown to provide the most stable antidepressant effect compared to non-selective COX-inhibitors. Suggested mechanisms behind the antidepressant effects were attenuation of neuroinflammation, HPA-axis dysregulation and altered monoamine expression.

## 1 Introduction

Depression is a common psychiatric disease with high personal and socio-economical costs. Traditionally, monoamines have been considered to play a significant role in the disease and therefore, most antidepressant treatments target these neurotransmitter systems. However, only approximately half of the patients experience sufficient symptom relief. This indicates that additional biological mechanisms play a role in the aetiology of depression (Williams et al., 2010). More and more evidence point to the role of inflammation in the pathophysiology of the disease. It is known that cancer patients without psychiatric history who receive treatment with interferons are at increased risk of developing depression (Capuron et al., 2000; Raison et al., 2005). Furthermore, depressive symptoms appear in animal models when cytokine levels are experimentally elevated, for example, by injections of the bacterial endotoxin lipopolysaccharide (Bay-Richter et al., 2011) or by poly I:C, which simulates a viral infection (Gibney et al., 2013). Interestingly, depressed patients have, in some studies, been shown to have elevated plasma levels of pro-inflammatory cytokines (Hestad et al., 2003), and it has been suggested that cytokine levels correlate with the severity of depression (Sha et al., 2022). Furthermore, the HPA axis is known to be dysregulated in depression. The negative cortisol feedback to the hypothalamus, pituitary and immune system is impaired. This leads to continual activation of the HPA axis and excess cortisol release. Cortisol receptors become desensitized, leading to increased activity of the pro-inflammatory immune mediators and disturbances in neurotransmitter transmission. Approximately 50% of patients suffering from MDD experience elevated cortisol secretion (Blackburn-Munro and Blackburn-Munro, 2001). Interestingly, some studies have shown that traditional antidepressant drugs can reduce inflammation (Hannestad et al., 2011; Cattaneo et al., 2013).

Because of the above evidence, a natural next step would be to assess the antidepressant effects of anti-inflammatory drugs. Clinical trials have been performed to evaluate the efficacy of anti-inflammatory drugs on depressive symptomatology. Specifically, three double-blind, placebo controlled studies have been performed to examine the putative antidepressant effects of add-on treatment with celecoxib. All three studies showed a significant impact of celecoxib compared to monotherapy with the antidepressants, which were either reboxetine (a noradrenaline reuptake inhibitor) (Muller et al., 2006), fluoxetine (an SSRI) (Akhondzadeh et al., 2009), or sertraline (an SSRI) (Abbasi et al., 2012). Only a few clinical studies using NSAIDs as monotherapy have been performed. Of the few done, one study found that celecoxib, naproxen, and ibuprofen improve depressive symptomatology compared to placebo (Iyengar et al., 2013). On the contrary, Fields et al. (2012) found no effect of celecoxib or naproxen on depressive symptoms in persons above 70 years old. Similarly, Berk et al. (2020) found low-dose aspirin not to prevent depression in individuals older than 70 years old. A large randomised controlled trial failed to demonstrate an effect of minocycline and celecoxib on depressive symptoms in patients with bipolar depression (Husain et al., 2020). This significant heterogeneity of results has sparked debate as to the underlying course of the results. Why do anti-inflammatory drugs have an effect in some studies and not in others? It has, for example, been suggested that the anti-inflammatory treatment should only be applied in subgroups of patients who are known to have inflammation (Miller and Pariante, 2020).

NSAIDs exert anti-inflammatory effects by inhibiting pro-inflammatory cytokines through inhibitory effects on the COX-enzymes. Depending on chemical structure, the drug can be either non-selective for the COX-enzymes, have a preference for COX-2 or be selective for COX-2 exclusively. In the current review, we wish to present an overview of the preclinical results which exist so far using validated measures of depressive-like behaviour and NSAIDs as intervention. We will evaluate the importance of the depression model, animal species, sex, drug, dose, and treatment regimen. Furthermore, we provide an overview of the potential mechanisms for the antidepressant effects of NSAIDs, as highlighted in the included articles.

## 2 Materials and methods

### 2.1 Search Strategy and Selection Criteria

We searched the PubMed database for studies on the effects of NSAIDs on depressive-like behaviour in rodents. The search was performed on 28^th^ October 2021. Only primary articles published in peer-reviewed journals in English using an FDA approved NSAID and a validated measure of depressive-like behaviour such as forced swim test (FST)/tail suspension test (TST) or sucrose preference test (SPT). Opinion articles, commentaries, reviews, and other articles without original data were excluded.

### 2.2 Search String

We used a combined set of keywords to perform the PubMed search. These were: (“depres*”[Title/Abstract] OR “swim test”[Title/Abstract] OR “sucrose preference”[Title/Abstract] OR “porsolt”[Title/Abstract] OR “forced swim”[Title/Abstract] OR (“depressive disorder”[MeSH Terms] OR “depression”[MeSH Terms]) OR “depressive disorder”[MeSH Terms]) AND (“anti inflammatory drugs”[Title/Abstract] OR “anti inflammatory drug*”[Title/Abstract] OR “nsaid*”[Title/Abstract] OR “anti inflammatory agents, non steroidal”[MeSH Terms] OR “anti inflammatory agents”[MeSH Terms]) AND (“mouse”[Title/Abstract] OR “mice”[Title/Abstract] OR “rat”[Title/Abstract] OR “rats”[Title/Abstract] OR “muridae”[Title/Abstract] OR “mice”[MeSH Terms] OR “rats”[MeSH Terms]).

### 2.3 Study Selection and Data Extraction

Study selection and data extraction were performed using Covidence systematic review software (Veritas Health Innovation, Australia). Title/abstract screening and full-text screenings were performed by CBR. In cases of doubt during either title/abstract screening or full-text screening, the study was forwarded to a second reviewer (GW), who made the final decision. We included rodent studies that used an FDA approved NSAID to reverse depressive-like behaviour. The depressive-like behaviour could be produced by the behavioural test itself (e.g., FST) or by a range of inducers, including genetic models, inflammation and stress. We excluded studies using anti-inflammatory drugs that did not belong to the NSAID class or were not FDA approved. Furthermore, studies were excluded when the induction of depression was unclear (for example, in cases where immobility in the FST was confounded by general hypolocomotion). Additionally, the included studies had to contain a behavioural outcome measure of depression. Following descriptive variables were extracted for each study and presented in Supplementary Table S1: Reference, Primary aim of the study, Sex, strain and species, Age and/or bodyweight, Depression model, Pharmacological intervention, and Main findings.

## 3 Results

**FIGURE 1:**
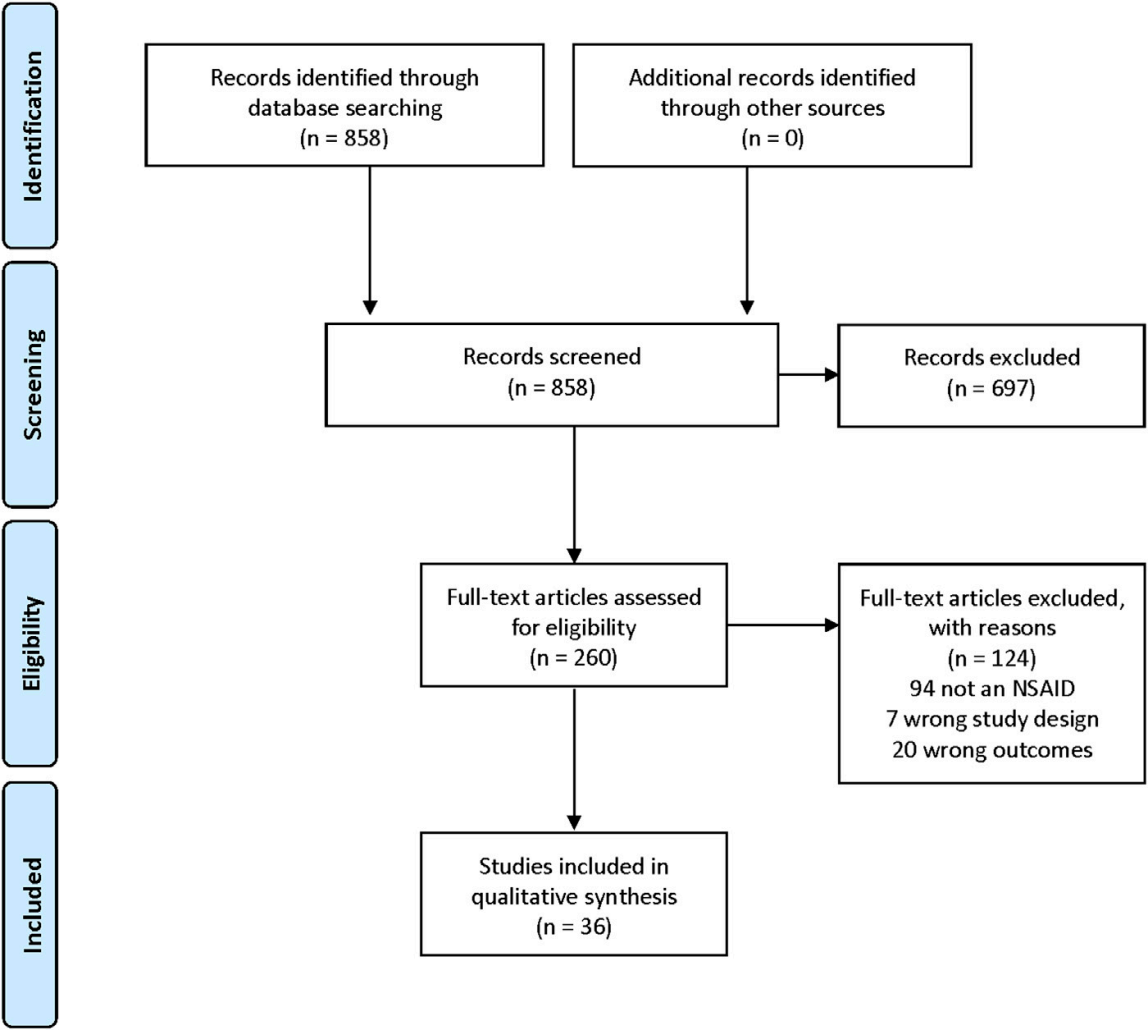
Study selection (PRISMA flow chart).

The search resulted in 858 references. Of these, 697 were excluded as irrelevant during the title-abstract screening. Mostly these references used a pharmaceutical intervention that was not classified as an NSAID. 260 articles were full-text screened. Of these, 124 were excluded; 94 because the intervention was not an NSAID, 7 had a wrong study design (e.g., where depressive-like behaviour could not be differentiated from locomotor abnormalities), and 20 had wrong outcomes (most often, no behavioural measure of depressive-like behaviour). In total, 36 references were included in this review (Supplementary Table S1). The study selection process is illustrated in Figure 1. The extracted articles contain data on both rat and mouse studies. 13 studies used Sprague-Dawley (S-D) rats, 7 Wistar, 1 study used HIV-1 rats and 17 studies used different mouse strains, both inbred, outbred and genetically modified. Only three studies reported the use of female animals. The depression models used to induce a depressive state ranged from stress models [e.g., chronic mild stress (CMS)] to pain models (e.g. CFA injections), to inflammation models (e.g. LPS) to a range of genetic models (e.g. HIV-1 rats) and diet-related models. Also, the FST alone in some studies served to detect antidepressant activity. The depression models were primarily stress-related (17 studies) or inflammation-related (9 studies). The remaining 11 studies used models of pain, neurodegeneration, diet, somatic disease or chemotherapy. 17 different NSAIDs were used, belonging to several NSAID groups. Most commonly used were the selective COX-2 inhibitor celecoxib (11 studies) and the non-selective COX inhibitor ibuprofen (8 studies). Drugs were administered in different doses, for different lengths of time and using different routes of administration (most commonly p.o. or i.p.). The results are presented according to the chemical classification of the NSAID.

### 3.1 Carboxylic Acids

The carboxylic acids include salicylates (e.g., acetylsalicylic acid), fenamates (e.g., mefenamic acid), indole acetates (e.g., indomethacin), phenylacetates (e.g., diclofenac) and the propionates (e.g., ibuprofen). Typical of these NSAIDs is that they are non-selective COX-inhibitors.

#### 3.1.1 Propionates

Ibuprofen was used in 8 studies. One study found no effect on lupus-induced despair. Here, ibuprofen was administered to MRL-lpr mice in food for 14 weeks (Ballok et al., 2006). One study found ibuprofen to have an antidepressant-like effect on FST-induced despair (50 but not 75 mg/kg) as well as reducing interferon (IFN)-α induced despair and anhedonia in male mice (Mesripour et al., 2019). Another study found ibuprofen not to affect FST-induced despair but indeed to decrease tumour induced despair using female mice (Norden et al., 2015). Qadeer et al. (2018) found no effect on FST induced despair in Wistar rats but significantly decreased stress-induced distress. BCG-induced despair was also shown to be reduced in mice (Saleh et al., 2014). Salmani et al. (2021) shoved that ibuprofen decreased FST induced despair but had no effect on LPS-induced despair in male BALB/c mice. Seo et al. (2019) found ibuprofen to reduce stress-induced despair. When co-administered, Warner-Schmidt et al. (2011) reported that ibuprofen reversed the antidepressant effects of several traditional antidepressant drugs.

Naproxen was used in 2 studies. Both studies found no effect on FST induced despair in female and male C57BL/6 mice (Warner-Schmidt et al., 2011; Pavlock et al., 2021). Warner-Schmidt et al. (2011) even showed that naproxen reversed the antidepressant effects of citalopram.

One study examined the effect of ketoprofen and found it to reduce swim stress-induced despair (Guevara et al., 2015). Flurbiprofen did not add any antidepressant effect when co-administered with fluoxetine (Alboni et al., 2018).

#### 3.1.2 Indole Acetates

Five studies examined the effects of indomethacin. Deak et al. (2005) found no impact on FST-induced despair in male S-D rats. On the contrary Mesripour et al. (2019) found indomethacin to reverse FST-induced despair and improve IFN-α induced despair and anhedonia in male albino mice. Perveen et al. (2018) also found indomethacin to reverse FST induced despair and CMS induced despair in male S-D rats. Stachowicz (2020) reported indomethacin not to affect FST or TST induced despair but to improve the antidepressant effect of imipramine when given in combination in CD1 and C57/BL6 mice.

#### 3.1.3 Phenyl Acetates

Diclofenac was examined in three studies. Borges et al. (2014) found diclofenac to reduce monoarthritis-induced despair in male S-D rats, and De La Garza et al. (2005) showed the same for LPS induced despair in male Wistar rats. Perveen et al. (2018) reported that diclofenac reduced FST- and stress-induced despair in male S-D rats.

#### 3.1.4 Salicylates

Acetylsalicylic acid (ASA) was used in 4 studies. Alboni et al. (2018); Brunello et al. (2006) both showed that in co-administration with fluoxetine, ASA decreased stress-induced depressive-like behaviour in male S-D rats. Alone, ASA had no antidepressant effect (Brunello et al., 2006; Warner-Schmidt et al., 2011). On the contrary (Guan et al., 2014) found ASA monoadministration to have antidepressant effects in the FST in male S-D rats. Warner-Schmidt et al. (2011) even showed that ASA attenuated the antidepressant effects of citalopram.

#### 3.1.5 Fenamates

Mefenamic acid reduced CMS-induced anhedonia and despair in male C57/BL6 mice (Feng et al., 2020).

### 3.2 Diaryl Heterocyclic Compounds

Celecoxib was used in 10 studies and was, therefore, the most frequently used NSAID included in this review. Belonging to the same drug group was rofecoxib which was used in one study. Common to these drugs is their selectivity as specific inhibitors of the COX-2 enzyme.

Alboni et al. (2018) found a non-significant tendency of celecoxib to increase the antidepressant effects of fluoxetine when coadministered. de Munter et al. (2020) showed that celecoxib did not affect FST-induced despair in WT mice but in a genetic model of frontotemporal lobar degeneration (FUS[1-359]-tg mice) the drug reduced despair. There was also a tendency for celecoxib to reduce IFN-α induced despair in male S-D rats (Fischer et al., 2015). Guo et al. (2009) found celecoxib to reduce CMS-induced anhedonia in male S-D rats and Kurhe et al. (2014) showed reduced despair and anhedonia in a high-fat diet model of depression in swiss albino mice. Maciel et al. (2013) reported that celecoxib could reduce despair-like behaviour induced by peripheral inflammation and Mesripour et al. (2019) showed that celecoxib had antidepressant effects in FST and reduced IFN-α induced despair and anhedonia. Further, celecoxib reduced Aβ-induced despair (Morgese et al., 2018) and CMS-induced anhedonia in male Wistar rats (Santiago et al., 2014). In the same study, celecoxib was found to have antidepressant effects in the FST. Song et al. (2019) did not find antidepressant effects in the FST after celecoxib treatment but did show celecoxib to reduce stress- and LPS-induced despair. Rofecoxib had antidepressant effects in an Nrf2 KO model of depression (Martín-de-Saavedra et al., 2013).

### 3.3 Enolic Acid Derivatives

The enolic acid derivatives (oxicams) include both preferential COX-2 inhibitors such as meloxicam, but also non-selective COX-inhibitors such as lornoxicam and piroxicam. Four studies examined meloxicam. Meloxicam was reported to improve repeated swim stress-induced despair in male S-D rats and reduce CMS-induced anhedonia in S-D rats (Guevara et al., 2015; Luo et al., 2017). Nemeth and colleagues found meloxicam to improve microembolism-induced despair but not HIV-induced despair in rats (Nemeth et al., 2014; Nemeth et al., 2016). Santiago and colleagues found a single dose of piroxicam to reduce FST-induced despair. Further, the drug reversed CMS-induced anhedonia and 6-OHDA-induced despair and anhedonia in male Wistar rats (Santiago et al., 2014; Santiago et al., 2015). Lornoxicam did not affect neuropathic pain induced despair (Hu et al., 2010).

### 3.4 Sulphonanilides

The preferential COX-2 inhibitor nimesulide was used in two studies. Both studies found nimesulide to reverse stress-induced despair in male S-D rats and male albino Laca mice (Singh et al., 2017; Luo et al., 2020).

## 4 Discussion

In summary, we identified and included 36 studies which met the inclusion criteria. The studies included examinations of both rats and mice. Inbred, outbred as well as genetically modified animals were used. Antidepressant effects of the NSAIDs alone were examined in the FST and/or TST in some studies. Still, most studies examined whether an NSAID drug could reverse experimentally induced depressive-like behaviour.

### 4.1 Depression Model

Thirteen studies examined the antidepressant effects of the NSAID in the FST without further manipulations of the animals (Yamano et al., 2000; Deak et al., 2005; Warner-Schmidt et al., 2011; Guan et al., 2014; Santiago et al., 2014; Norden et al., 2015; Perveen et al., 2018; Qadeer et al., 2018; Mesripour et al., 2019; Song et al., 2019; Stachowicz, 2020; Pavlock et al., 2021; Salmani et al., 2021). ASA, Celecoxib, ibuprofen, piroxicam, and indomethacin were shown to have antidepressant effects (Guan et al., 2014; Santiago et al., 2014; Perveen et al., 2018; Mesripour et al., 2019; Salmani et al., 2021). Other studies showed no antidepressant effects in the FST of ibuprofen, celecoxib, indomethacin, ASA or naproxen (Yamano et al., 2000; Warner-Schmidt et al., 2011; Norden et al., 2015; Qadeer et al., 2018; Song et al., 2019; Stachowicz, 2020; Pavlock et al., 2021).

In all studies, independent of the depression model, 29 out of 36 reported antidepressant effects of NSAIDs. The models used were either stress-induced depressive-like behaviour (15 of 29) (Brunello et al., 2006; Guo et al., 2009; Guan et al., 2014; Santiago et al., 2014; Guevara et al., 2015; Luo et al., 2017; Singh et al., 2017; Alboni et al., 2018; Perveen et al., 2018; Qadeer et al., 2018; Seo et al., 2019; Song et al., 2019; Feng et al., 2020; Luo et al., 2020; Stachowicz, 2020), 5 studies were directly related to inflammation (De La Garza et al., 2005; Saleh et al., 2014; Fischer et al., 2015; Mesripour et al., 2019; Salmani et al., 2021), 8 studies used different disease models (Maciel et al., 2013; Martín-de-Saavedra et al., 2013; Borges et al., 2014; Norden et al., 2015; Santiago et al., 2015; Nemeth et al., 2016; Morgese et al., 2018; de Munter et al., 2020) and a single study used diet-induced depression (Kurhe et al., 2014). Six studies failed to find antidepressant effects of NSAIDs. The models used were either stress-induced (by FST) (Deak et al., 2005), inflammation-induced (Yamano et al., 2000) or related to a disease (Ballok et al., 2006; Hu et al., 2010; Nemeth et al., 2014; Pavlock et al., 2021). A single study found antidepressant drugs to reverse the antidepressant effects of several antidepressant drugs in mice (Warner-Schmidt et al., 2011).

Intriguingly, the only stress-related model which fails to find antidepressant effects of NSAIDs is the FST alone (Deak et al., 2005). Of the 29 studies reporting antidepressant effects, 13 use stress models, primarily CMS. All studies using CMS report antidepressant effects of the NSAIDs. Therefore, stress models, in particular CMS models, appear to be a good choice when examining the antidepressant effects of NSAIDs. This may also have clinical relevance; NSAIDs may be better at treating depression related to stress than other types of depressive illness. This could be important as more and more people suffer from stress-related depression, and this has severe socioeconomic as well as personal consequences (Yang et al., 2015).

### 4.2 Species and Sex

Rats were used in 21 studies, whereas 15 used different mouse strains. The most frequently used strain was the S-D rat (used in 13 studies). In the 12 studies which examined antidepressant effects in the FST mice and rats were test subjects both in the studies showing antidepressant activity of NSAIDs as well as studies failing to find an effect. Intriguingly, 5 out of 7 studies failing to find antidepressant effects of NSAIDs in the FST without other manipulations were performed on mice. This pattern is similar when looking at all models; Of the 29 studies showing antidepressant properties of NSAIDs, 11 studies used mice (38%). Out of the 7 studies that failed to find an antidepressant activity, 5 studies used mice (71%). The rat studies which failed to report antidepressant effects used the FST alone and a model of neuropathic pain. The mouse models without antidepressant effects used inflammation models, different disease models, and FST alone. As the same or similar models are also used in studies that report antidepressant effects of the NSAIDs, it seems unlikely that methodological differences cause the species difference. The same applies to the drugs used; several different classes of NSAIDs were used in both mice and rats (see Supplementary Table S1). In summary, it appears that rat models are better at detecting antidepressant properties using the FST. This is in line with previous research (Borsini and Meli, 1988).

Of the 6 studies failing to show antidepressant effects of NSAIDs, one study used IFN-α to induce a depressive state in mice. In this study, indomethacin could not reverse the depressive-like behaviour (Yamano et al., 2000). Interestingly, Mesripour et al. (2019) also studied the effects of indomethacin after IFN-α-induced depression. Here, indomethacin was able to reverse IFN-α-induced despair and anhedonia. The groups used different mouse strains; ddY mice vs. non-specified albino mice. While ddY is sometimes used as a general purpose model, it is also known that the mouse develops spontaneous IgA nephropathy (Imai et al., 1985), which could affect the result.

Intriguingly, all studies reporting the use of female animals failed to detect the antidepressant properties of NSAIDs. Preclinical work has traditionally been performed exclusively in male animals. Still, more and more research suggests that disease progression and therapeutic drug response may vary substantially between the sexes (see LeGates et al. (2019) for review). An important goal of future research will be to explore potential sex differences further, and future studies should therefore include both male and female animals when examining drug effects of e.g., NSAIDs.

### 4.3 Drug, Dose and Treatment Regiment

Two studies examined the effect of indomethacin on IFN-α—induced depression in mice and found conflicting evidence (Yamano et al., 2000; Mesripour et al., 2019). Apart from the different mouse strains used, as described above, the studies also used different doses, routes of administration and lengths of treatment. Yamano et al. (2000), who failed to find an effect of indomethacin, used 10 mg/kg, s.c. for 7 days Mesripour et al. (2019) found an effect of a single injection of 25 mg/kg i.p indomethacin 30 min before the FST. One explanation for the discrepancy could therefore be the chosen dose. It should, however, be noted that both Perveen et al. (2018) also reported on effects of indomethacin on stress-induced despair. Here, 7 days of treatment with 7.5 mg/kg indomethacin had antidepressant properties in the FST in male rats (Perveen et al., 2018), whereas, for mice, 2 mg/kg indomethacin for 7 or 14 days was not able to produce antidepressant effects in the FST (Stachowicz, 2020). Mice likely require a larger dose than rats as smaller animals have higher metabolic rates and higher physiological processes (Nair and Jacob, 2016).

Surprisingly, Warner-Schmidt et al. (2011) reported that ibuprofen reversed the antidepressant effects of citalopram, fluoxetine, imipramine, and desipramine and that naproxen and ASA reversed the effect of citalopram in C57Bl6 mice. The only other included study examining the effect of NSAIDs and antidepressants on antidepressant effects was (Stachowicz, 2020), who reported that indomethacin improved the antidepressant effect of imipramine when administered together. A difference between the two studies is the use of different NSAIDs. Where ibuprofen is a propriate, indomethacin belongs to the indole acetates.

Interestingly, all studies applying selective COX-2 inhibitors (celecoxib and rofecoxib) reported antidepressant effects of these drugs (Guo et al., 2009; Maciel et al., 2013; Martín-de-Saavedra et al., 2013; Kurhe et al., 2014; Santiago et al., 2014; Fischer et al., 2015; Alboni et al., 2018; Morgese et al., 2018; Mesripour et al., 2019; Song et al., 2019; de Munter et al., 2020; Feng et al., 2020). It has previously been reported that selective COX-2 inhibitors may be more effective in relieving depression than non-selective COX-inhibitors (Baune, 2017).

### 4.4 Mechanisms

In many of the studies, neurobiological mechanisms which may underlie the antidepressant effects of the NSAID are examined. Eight studies which reported antidepressant effects of NSAIDs have examined neuroinflammation in animals. Five of these report that NSAIDs lead to decreased neuroinflammation (Maciel et al., 2013; Norden et al., 2015; Song et al., 2019; de Munter et al., 2020; Feng et al., 2020) either measured as normalisation of microgliosis or cytokine expression in the brain. De La Garza et al. (2005) and Guan et al. (2014) examined peripheral cytokines but reported conflicting results. Guan et al. (2014) found blood levels of TNF-a and IL-6 normalised after ASA treatment, whereas De La Garza et al. (2005) found no effect of diclofenac on LPS-induced elevation of plasma IL-1β. Of the studies which failed to find antidepressant effects of NSAIDs, three examined neuroinflammation. Ballok et al. (2006) reported that ibuprofen neither affects lupus-induced despair nor microgliosis. For this study, it should be noted that ibuprofen is provided in the food, and no measure of the actual dose is reported. Nemeth et al. (2014) and Warner-Schmidt et al. (2011) showed that neuroinflammation is decreased by meloxicam and ibuprofen without having an antidepressant effect. It therefore appears that reversal of neuroinflammation could be an essential player in the anti-inflammatory effects of NSAIDs, as has often been suggested (Leonard and Song, 1999; Hestad et al., 2003; Thomas et al., 2005), but caution should be taken before drawing such conclusion.

As described in the introduction, depression is often associated with dysregulation of the HPA-axis, which is linked to neuroinflammation (Holsboer, 2003), and several preclinical studies have shown that depressive-like behaviour is associated with elevated levels of CORT (McEwen, 2005; Pariante and Lightman, 2008). CORT expression was examined in four of the studies reporting antidepressant effects of NSAIDs. Three of these showed that meloxicam, ibuprofen, indomethacin and diclofenac normalised plasma CORT levels (Guevara et al., 2015; Perveen et al., 2018; Seo et al., 2019). Guan et al. (2014) reported that while ASA had antidepressant effects, the drug did not attenuate CORT levels. None of the studies which failed to find antidepressant properties of NSAIDs measured CORT. In summary, there is some evidence that CORT plays a role in the antidepressant activity of NSAIDs.

Monoamines are the main targets of classical antidepressants. Here, three studies reported how piroxicam and celecoxib could reverse both despair but also normalise the monoamine expression in the brain (Santiago et al., 2014; Morgese et al., 2018).

## 5 Conclusion

In summary, we found that antidepressant effects of NSAIDs was studied in several different depression models, using both mouse- and rat strains but primarily using male animals. Seventeen different NSAIDs were examined for potential antidepressant effects. The results showed that stress models using selective COX-2 inhibitors provided the most robust antidepressant response. This may have clinical implications as it could be speculated that patients with stress-related depression are more likely to benefit from NSAID treatment than other types of depression and that the most efficient treatment would be selective COX-2 inhibitors such as celecoxib and rofecoxib.

## Data Availability

The original contributions presented in the study are included in the article/Supplementary Material, further inquiries can be directed to the corresponding author.
