# Supplementary material for: Antidepressant Effects of NSAIDs in Rodent Models of Depression—A Systematic Review
Source: Front Pharmacol. 2022 Jun 8;13:909981. doi: 10.3389/fphar.2022.909981 (PMC9213814; doi:10.3389/fphar.2022.909981)
Supplement: Supplementary file 1 [file Table1.pdf]

| Reference (setting) | Primary aim of study | Sex, strain and species (n/group) | Age and/or bodyweight | Depression model | Pharmacological intervention (dose, route of administration) | Main findings |
|---|---|---|---|---|---|---|
| Alboni et al., 2018 (Italy) | Examination of NSAIDs ability to increase effectiveness of antidepressants | Male S-D rats (5-10) | 200-250g | Minor unavoidable stress | FLX (5mg/kg, i.p) + Flurbiprofen (5mg/kg, i.g)<br>FLX (5mg/kg, i.p)+ Celecoxib (5mg/kg, i.g)<br>FLX (5mg/kg, i.p)+ ASA (11.25-45mg/kg, i.p)<br>7 days of treatment | FLX + ASA → ↓escape deficit.Amplitude of antidepressant effect of ASA is dose dependant<br>FLX + celecoxib → (↓) dep<br>FLX + flurbiprofen → Ø |
| Ballok et al., 2006 (Canada) | Examination of ibuprofen's effects on lupus-induced brain atrophy and behavioural dysfunction | Male MRL-lpr mice (10) | 5-19 weeks | MRL-lpr lupus model | Ibuprofen (375 ppm in food chow) 14 weeks | Ibuprofen → no effect on lupus induced despair or on lupus induced microgliosis |
| Borges et al., 2014 (Spain) | To examine whether NSAIDs can alleviate arthritic pain induced anxiety and [illegible] | Male S-D rats (5-6) | 250-300g | MoA induced by CFA injection | Diclofenac (10mg topically) twice daily for 3-5 days | Diclofenac → ↓MoA induced despair and restored ERK1/2 activation levels in the brain |
| Brunello et al., 2006 (Italy) | Evaluate the antidepressant effect of coadministation with ASA and FLX | Male S-D rats (7-8) | 150-175 g | Chronic escape deficit model | FLX (5mg/kg, i.p)<br>ASA (22.5 or 45mg/kg, i.p)<br>testing after 1, 2 and 3 weeks of treatment | FLX + ASA → ↓escape deficit<br>FLX → ↓escape deficit<br>ASA → Ø<br>Faster therapeutic effect of FLX + ASA compared to FLX alone |
| De La Garza II 2005 (USA) | To examine the effect of diclofenac on LPS-induced anhedonia | Male Wistar rats (8-9) | 8 weeks, 225-250g | LPS | Diclofenac (2.5mg/kg, s.c) 2.5 h before testing<br>LPS (20ug/kg, i.p) 2h before testing | Diclofenac → ↓LPS-induced anhedonia but had no effect on LPS-induced elevation of plasma IL-1β |

| | | | | | | |
|---|---|---|---|---|---|---|
| de Munter et al., 2020 (Russia/Netherlands) | To examine abnormal behaviour in a genetic model of frontotemporal lobar degeneration (FUS[1-359]-tg mice) | Male FUS[1-359]-tg mice (9-15) | 9-10 weeks | FUS[1-359]-tg mice | Celecoxib (30mg/kg/day, p.o) 3 weeks treatment | Celecoxib in WT → Ø despair<br>Celecoxib in FUS[1-359]-tg → ↓despair and normalized IL-1B expression and microgliosis in the brain |
| Deak et al., 2005 (USA) | To examine the effects of anti-inflammatory agents and LPS on FST | male S-D rats (8) | 350-400g | FST | Indomethacin (1 or 10mg/kg, i.p), 60 min before FST | Indomethacin → Ø |
| Feng et al., 2020 (China) | To examine effects of MA and celecoxib on depressive-like behaviour and microgliosis | Male C57BL/6 mice (8) | 7 weeks, 20-25g | 28 day CMS | MA (5mg/kg/day, i.p)<br>celecoxib (10mg/kg/day, i.p)<br>28 days treatment | MA →↓CMS induced anhedonia, despair and microgliosis<br>Celecoxib→↓CMS induced anhedonia, despair and microgliosis. MA and celecoxib inhibit ERK1/2 and P38 MAPK activation and iNOS expression. |
| Fischer et al., 2015 (Denmark) | To investigate of IFN-α on depression and TRP metabolites | Male S-D rats (10) | 300g | IFN-α (60000 IU/kg/day), 7 days | Celecoxib (16mg/kg/day, p.o), 7 days treatment | Celecoxib → (↓)INF-α induced despair and altered KYN/TRP ratio |
| Guan et al., 2014 (China) | To investigate antidepressant effects of ASA | Male S-D rats (7) | 220-250g | FST | ASA (6, 12, 25 and 50mg/kg, i.p) treatment 24, 5 and 1 h before FST | ASA (25 and 50mg/kg) → ↓FST-induced depression and normalised peripheral TNF-α and IL-6 levels but had no effect on FST-induced CORT |
| Guevara et al., 2015 (Venezuela) | To evaluate the association between spinal PGE2 and thermal hyperalgesia following stress. | Male S-D rats (?) | 150-300g | Repeated swim stress | Ketoprofen (6mg/kg, i.p)<br>Meloxicam (1mg/kg, i.p) treatment 30 min before swim session (3 sessions in total) | Ketoprofen → ↓repeated swim test induced despair<br>Meloxicam → ↓repeated swim test induced despair. Ketoprofen and meloxicam reduced serum CORT and normalised inflammation induced spinal [illegible] |
| Guo et al., 2009 (China) | To examine the antidepressant effects of celexocib | Male S-D rats (?) | 200-220g | CMS | Celecoxib (2, 8 or 16mg/kg/day, p.o) 21 days<br>Celecoxib (16mg/kg, p.o.) single dose<br>1 h before stress exposure | Celecoxib (8 and 16 mg/kg/day) → ↓CMS induced anhedonia and brain COX-2 and PGE2 elevation |

| | | | | | | |
|---|---|---|---|---|---|---|
| Hu et al., 2010 (China) | To examine whether neuropathic pain can induce cognitive dysfunction | Male S-D rats (10) | 160-180g | L5 spinal nerve transection | Lornoxicam (2 mg/kg/day, i.p) on postoperative days 7-28 | Lornoxicam → no effect on neuropathic pain induced despair |
| Kurhe et al., 2014 (India) | To examine if celexocib has antidepressant effects on obese mice | Male Swiss albino mice (6) | 20-25g | HFD | Celexocib (10mg/kg/day, p.o) 28 days | Celecoxib → ↓HFD induced despair and anhedonia |
| Luo et al., 2017 (China) | To examine the role of COX-2 in learning and memory [illegible] | ? S-D rats (10) | 8 weeks, 180-200g | CMS | Meloxicam (3mg/kg/day, p.o.) 21 days | Meloxicam → ↓CMS-induced anhedonia |
| Luo et al., 2020 (China) | To screen target proteins regulated by nimesulide in hippocampus of CUMS rats | Male S-D rats (20) | 180-220g | CMS | Nimesulide (12mg/kg/day, p.o) 21 days | Nimesulide → ↓CMS-induced despair and neuroinflammation |
| Maciel et al., 2013 (Brazil) | To characterize depression-like behaviour in an inflammation model induced by CFA | Male Swiss mice (6-8) | 25-30g | CFA | Celecoxib (3, 15 or 30 mg/kg/day p.o) 7 days | Celecoxib (15 and 30mg/kg) → ↓CFA-induced despair and neuroinflammation |
| Martin-de-Saavedra et al., 2013 (Spain) | To examine the role of Nrf2 in depression | Male C57Bl/6 mice Nrf2 WT and KO (10) | 3-4 months | Nrf2 knockout | Rofecoxib (2mg/kg/day i.p) 7 days | Rofecoxib → ↓Nrf2 KO-induced despair |

| | | | | | | |
|---|---|---|---|---|---|---|
| Mesripour et al., 2019 (Iran) | To examine the effects of NSAIDs on IFN-α induced depression | Male albino mice (6) | 24-28g | IFN-α (16 x 10^5 IU/kg, s.c) 6 days | Celecoxib (25 or 50 mg/kg/day i.p)<br>Ibuprofen (50 or 75 mg/kg/day i.p)<br>Indomethacin (12.5 or 25 mg/kg/day i.p)<br>1 or 6 days of treatment | Celecoxib (50 but not 25mg/kg) → antidep effect alone in FST<br>Celecoxib (25 and 50mg/kg) → ↓IFN-α induced despair<br>Celecoxib (50 acute not 25 subacute) → ↓IFN-α induced anhedonia<br>Ibuprofen (50 but not 75mg/kg) → antidep effect alone in FST<br>Ibuprofen (50 acute and subacute) → ↓IFN-α induced despair and anhedonia<br>Indomethacin (12.5 and 25 mg/kg) → antidep effect alone in FST<br>Indomethacin (25mg/kg acute) → ↓IFN-α induced despair and anhedonia |
| Morgese et al., 2018 (Italy) | To examine the effects of celecoxib on Aβ induced [illegible] | Male Wistar rats (8) | 275-300g | ICV Aβ | Celecoxib (15mg/kg/day s.c.) 8 days | Celecoxib → ↓Aβ-induced despair and normalise 5HT levels |
| Nemeth et al., 2014 (USA) | To examine the effect of meloxicam on HIV-induced depression | Female WT and HIV-1 transgenic rats (5-9) | 48-58 days | HIV-1 | Meloxicam (1mg/kg/day, p.o) 33 days | Meloxicam → no effect on HIV-induced despair but prevents neuroinflammation |
| Nemeth et al., 2016 (USA) | To examine inflammatory mechanism in microembolism-induced depressive-like behaviours | Male Wistar rats (12-13) | 3 months | Microembolism | Meloxicam (1mg/kg/day i.p) 14 days | Meloxicam → ↓microembolism-induced anhedonia and normalise expression of Spp1 |
| Norden et al., 2015 (USA) | To examine the effect of ibuprofen on cancer induced behavioural alterations | Female BALB/c x DBA/2 F1 (CD2F1) mice (?) | 10 weeks 20-22g | Cancer tumor | Ibuprofen (10mg/kg/day p.o) 10 days | Ibuprofen → ↓tumor-induced despair and peripheral + central inflammation<br>Ibuprofen alone → no effect on despair |

| | | | | | | |
|---|---|---|---|---|---|---|
| Pavlock et al., 2021 (USA) | To examine neuroinflammation in OVX mice treated with the chemotherapy drug [illegible] | Female C57Bl/6 mice (6-8) | 9-10 weeks, 16-20g | CP | Naproxen (375 ppm in diet) 3 weeks | Naproxen → no effect on despair neither alone nor in OVX/CP animals |
| Perveen et al., 2018 (Pakistan) | To examine the role of COX-2 inhibitors on stress-induced behavioural [illegible] | Male S-D rats (6) | 150-200g | 7 days mild stress | Indomethacin (7.5mg/kg/day, i.p)<br>Diclofenac (5 mg/kg/day, i.p)<br>[illegible] | Indomethacin → ↓despair alone and after stress and normalised plasma CORT levels<br>Diclofenac → ↓despair alone and after stress and normalised plasma CORT levels |
| Qadeer et al., 2018 (Pakistan) | To examine the role of ibuprofen and lavender oil on stress induced behavioural abnormalities | ? Wistar rats (6) | 150-200 | Restraint stress | Ibuprofen (2mg/kg/day p.o) 7 days of treatment | Ibuprofen → ↓stress-induced despair. No effect in unstressed animals |
| Saleh et al., 2014 (Egypt) | To examine the effect of ibuprofen in BCG induced depressive-like behaviour | Male Swiss albino mice (7-10) | 22-30g | BCG | Ibuprofen (40mg/kg/day p.o) 2 weeks treatment | Ibuprofen → ↓BCG induced despair in both TST and FST |
| Salmani et al., 2020 (Iran) | To examine the effects of ibuprofen on LPS induced brain alterations | Male BALB/c mice (10-11) | 2 months, 25-35g | LPS (250ug/kg/day, 6 days) | Ibuprofen (40mg/kg/day i.p) 10 days treatment | Ibuprofen+vehicle → ↓despair<br>Ibuprofen+LPS → Ø despair but decreased neuroinflammation |
| Santiago et al., 2014 (Brazil) | To examine the antidepressant-like effects of celecoxib and piroxicam | Male Wistar rats (8-11) | 3 months, 280-320g | 3 weeks CMS | Celecoxib (10mg/kg p.o)<br>Piroxicam (10mg/kg p.o)<br>Treatment 1, 5 and 24h before testing or daily for 21 days | Celecoxib alone → ↓despair and altered monoamine expression<br>Piroxicam alone → ↓despair and altered monoamine expression<br>Celecoxib → ↓CMS-induced anhedonia<br>Pirocixam → ↓CMS-induced anhedonia |
| Santiago et al., 2015 (Brazil) | To investigate the antidepressant effects of piroxicam on 6-OHDA lessioned [illegible] | Male Wistar rats (6-9) | 280-320g | 6-OHDA lessions of substantia nigra | Piroxicam (10mg/kg/day p.o) 21 day treatment | Piroxicam → ↓6-OHDA induced despair and anhedonia and normalized monoamine levels |

| | | | | | | |
|---|---|---|---|---|---|---|
| Seo et al., 2019 (Korea) | To examine the effect of escitalopram and ibuprofen on stress-induced behaviour | Male S-D rats (12) | Adult | Chronic restraint stress (6h/day, 21 days) | Ibuprofen (40mg/kg/day i.p) 21 days | Ibuprofen → ↓stress-induced despair and normalised CORT and BDNF levels |
| Singh et al., 2017 (India) | To examine protective effects of losartan and ramipril on stress induced insulin resistance | Male albino Laca mice (6) | 4-6 months, 20-30g | 30 days Chronic restraint stress | Nimesulide (10mg/kg/day p.o), 30 days<br>Losartan (20mg/kg/day), 30 days | Nimesulide → ↓stress induced despair<br>Nimesulide + Losartan → ↓stress induced despair |
| Song et al., 2019 (China) | To examine the effect of celecoxib on neuronal injury on a rat model of depression | Male Wistar rats (18) | 5-6 weeks, 160-180g | 5 weeks CMS or 0.5 mg/kg/day LPS 7 days | Celecoxib (20mg/kg/day, i.p) 5 weeks treatment | Celecoxib → ↓stress-induced and LPS-induced anhedonia and despair and normalised brain PGE2 and cytokine expression<br>Celecoxib → Ø on anhedonia and despair in control rats |
| Stachowicz 2020 (Poland) | To examine if indomethacin interacts with MTEP in producing antidepressant-like activity | ? CD1 and C57Bl/6 mice (6-7) | ? | FST or TST | Indomethacin (2 mg/kg/day i.p) for 1, 7 or 14 days<br>Imipramine (10 mg/kg/day i.p) for 1, 7 or 14 days | Imipramine + indomethacin → ↓FST induced despair (14 but not 7 days of treatment, CD1)<br>Imipramine + indomethacin → ↓TST induced despair (1 and 7 days of treatment, C57Bl/6)<br>Indomethacin → Ø FST induced despair (7 and 14 days of treatment, CD1)<br>Imipramine → Ø FST induced despair (7 and 14 days of treatment, CD1) |
| Warner-Schmidt et al., 2011 (USA) | To investigate a possible interaction between antidepressant agents and antiinflammatory drugs on depression related behaviour | Male C57BL/6 mice (8-16) | 8-10 weeks | FST and TST | Ibuprofen (70 mg/kg/day p.o)<br>Naproxen (140 mg/kg/day p.o)<br>Acetylsalicylic acid (210 mg/kg/day p.o)<br>5-7 days treatment | The NSAIDs alone did not affect despair-like behaviour. Ibuprofen reversed antidepressant effects of citalopram, FLX, imipramine, desipramine but not tranylcipromine or bupropion in FST and TST.<br>Naproxen and acetylsalicylic acid also reversed the antidepressant effects of citalopram.<br>Ibuprofen, naproxen, acetylsalicylic acid and acetaminophen did not have an effect on TST or FST behaviour. Ibuprofen decreased brain IL-1β |

| | | | | | | |
|---|---|---|---|---|---|---|
| Yamano et al., 1999 (Japan) | To investigate the mechanism of IFN-α induced depression | Male ddY mice (20) | 23-36 g | IFN-α (YM643 or sumiferon) | Indomethanin (10 mg/kg, s.c), 7 days | Indomethanin → Ø TST<br>Indomethanin → Ø in IFN-α induced despair |

Table 1. Studies examining antidepressant effects of NSAIDs using rodent models

Abbreviations: Significant decrease (↓); Non-significant decrease ((↓)); Non-steroidal antiinflammatory drug (NSAID); Sprague-Dawley (S-D); Fluoxetine (FLX); Acetylsalisylic acid (ASA); No effect (Ø); Monoarthritis (MoA); Complete Freund's adjuvant (CFA); Extracellular signal-regulated kinases 1 and 2 (ERK1/2); Lipopolysaccharide (LPS); Forced swim test (FST); Wildtype (WT); Knockout (KO); Chronic mild stress (CMS);Mefenamic acid (MA); Kynurenine (KYN); Tryptophan (TRP); Not reported (?); Prostaglandin E2 (PGE2); Corticosterone (CORT); High fat diet (HFD); Interferon (IFN); Interleukin (IL); Amyloyd beta (Aβ); Intracerebroventricular (ICV); Secreted phosphoprotein 1 (Spp1); Cyclophosphamide (CP); Ovariectomized (OVX); Cyclooxogenase (COX); Bacillus Calmette-Guerin (BCG); Tail suspension test (TST); 6-Hydroxydopamine (6-OHDA); Brain-derived neurotrophic factor (BDNF); Intraperitoneal (i.p); Intragastric
